# A reduced aperture allows for transcranial focus localization at lower pressure

**DOI:** 10.1121/10.0011695

**Published:** 2022-06-28

**Authors:** M. Anthony Phipps, Sumeeth Jonathan, Pai-Feng Yang, Li Min Chen, William Grissom, Charles F. Caskey

**Affiliations:** 1 Vanderbilt University Institute of Imaging Science, Vanderbilt University Medical Center, Nashville, Tennessee 37232, USA; 2 Biomedical Engineering, Vanderbilt University, Nashville, Tennessee 37232, USA m.anthony.phipps@vumc.org, sumeeth.v.jonathan@vanderbilt.edu, pai-feng.yang@vumc.org, limin.chen@vumc.org, will.grissom@vanderbilt.edu, charles.f.caskey@vumc.org

## Abstract

Localizing the focus during transcranial focused ultrasound procedures is important to ensure accurate targeting of specific brain regions and interpretation of results. Magnetic resonance acoustic radiation force imaging uses the displacement induced by the ultrasound focus in the brain to localize the beam, but the high pressure required to displace brain tissue may cause damage or confounds during subsequent neuromodulatory experiments. Here, reduced apertures were applied to a phased array transducer to generate comparable displacement to the full aperture but with 20% lower free field pressure.

## 1. Introduction

Transcranial focused ultrasound (FUS) is being widely explored for various uses. Thermal ablation of the thalamus with FUS is being used clinically to treat essential tremor (Elias *et al.*, 2016) and explored for other clinical conditions such as Alzheimer's disease (Costa e Souza *et al.*, 2018). The combination of FUS and microbubbles can be used to disrupt the blood–brain barrier (BBB) to allow drug delivery for cancer therapy and research (Burgess and Hynynen, 2013; Pouliopoulos *et al.*, 2020). Transcranial ultrasound stimulation for neuromodulation is being studied in small and large animals and humans (Blackmore *et al.*, 2019; Kamimura *et al.*, 2020). All use cases of transcranial FUS rely on accurate targeting of the FUS beam.

Measuring an effect on the tissue of the FUS beam provides the best means of estimating the focus location for transcranial studies. FUS can produce both thermal and mechanical bioeffects (O'Brien, 2007) that can be measured with magnetic resonance imaging (MRI). Temperature mapping with MRI (Marx *et al.*, 2017) can be used to measure heating changes within the brain to determine where the focus is located. Heating is induced by energy absorption in the tissue and can be used to assess an intensity profile of the beam. Typically, long (>10 s) continuous wave FUS pulses with relatively low intensity are used to generate heating. However, even relatively small temperature changes (0.5 °C–2 °C) that do not induce damage have been associated with changes in brain activity, possibly confounding experiments that attempt to use ultrasound for neural therapy or modulation (Wang *et al.*, 2014). In a study of albumin positive cells in a rat brain, BBB permeability was increased when the temperature was hyperthermic, 38 °C–42.5 °C (Kiyatkin and Sharma, 2009), and a thermal mechanism has been proposed for FUS neuromodulation (Darrow *et al.*, 2020). A method to estimate the location of an acoustic focus during transcranial ultrasound that does not rely on generating heat would be desirable.

Another method of estimating the ultrasound field during transcranial ultrasound applications is by measuring displacements that arise due to the acoustic radiation force—a force generated in the direction of propagation that arises due to transfer of momentum into the propagating medium. Magnetic resonance acoustic radiation force imaging (MR-ARFI) (Mougenot *et al.*, 2016a; Paquin *et al.*, 2013; Sarvazyan *et al.*, 1998; Zheng *et al.*, 2018) uses motion encoding gradients (MEGs) to measure phase changes caused by micron scale displacements between images with and without FUS being applied. Although MR-ARFI in phantoms is sensitive to displacements less than 100 nm, most *in vivo* cases require radiation forces capable of generating displacements >1 μm, which can be challenging to do within the safety limits of diagnostic ultrasound. The radiation force is proportional to intensity (often quoted as F=2αI/c for a propagating plane wave (Nightingale, 2011), where *F* is the radiation force, α is the absorption, *I* is the temporal average intensity, and *c* is the speed of sound). The absorption increases with increased ultrasound frequency, resulting in a larger force at higher pressures.

To generate a displacement greater than 1 μm in the brain at frequencies used for transcranial ultrasound (typically <1MHz), pressure levels are required that encroach on or exceed safety guidelines of diagnostic ultrasound. However, it is still important to minimize the energy input to the skull and brain. Optimizing the sensitivity of MR-ARFI can improve the ability to detect small displacements that use less energy, which has been demonstrated using bipolar MEGs (Chen *et al.*, 2010), optimizing the timing of the FUS pulses (Mougenot *et al.*, 2016b), and optimizing the orientation of the encoding gradients (Phipps *et al.*, 2019). Alternatively, achieving similar displacements with lower FUS intensities can also be used to minimize risk during beam localization. Previous work has shown that cavitation is more likely with larger *f*-numbers, suggesting nonlinearity is increased (Khokhlova *et al.*, 2018). This finding suggests that larger *f*-number transducers produce more nonlinear content. Since ARF scales with frequency, larger *f*-number transducers producing nonlinearities resulting in frequency harmonics should generate greater displacement. Additionally, by electronically reducing the aperture of a phased array therapy transducer, thus increasing the *f*-number, the remaining elements are positioned better geometrically so that a normal vector from the element is more aligned with the propagation direction (Wu, 1995). This geometric advantage combined with increased nonlinearity can lead to increased displacement for the same energy.

Here, we demonstrate the effects of ultrasound transducer *f*-number on pressure needed to acquire MR-ARFI displacement maps. We first performed water tank measurements and simulations to measure changes to the focal pattern with different *f*-numbers, calibrated the pressure output for each *f*-number configuration, and determined the nonlinear component of each *f*-number. Phantom studies were then carried out in a clinical MR scanner to determine the feasibility of these methods, and a set of *in vivo* scans was acquired. We demonstrate that the FUS beam can be localized with less peak negative pressure (PNP) compared to using the full aperture, allowing for a better safety profile and reducing the chance of unintended effects that could confound a treatment or experiment.

## 2. Materials and methods

### 2.1 Transducer time series and calibration

**Fig. 1.**
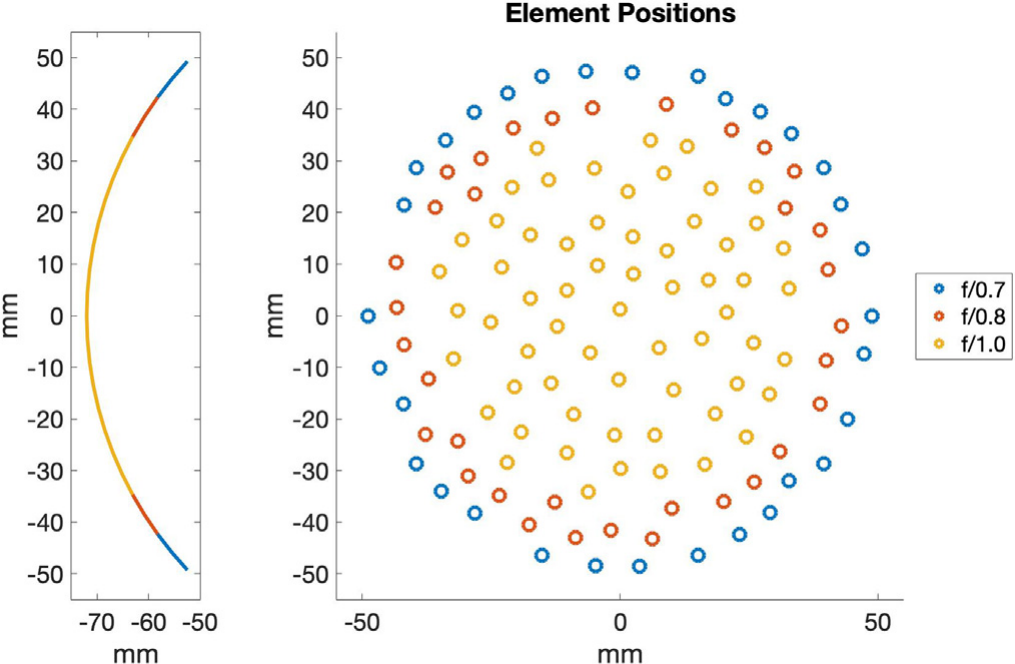
Left: A side profile of the transducer showing the decreasing aperture as elements are turned off and the *f*-number is increased. The full array has an *f*-number of 0.7 and 128 elements. By electronically turning off the outside set of elements (in blue), the *f*-number is increased to 0.8, and by further turning off the elements in red, the *f*-number is 1.0. Right: Element positions on the transducer showing which elements were turned off for each *f*-number configuration.

A spherically focused, 650 kHz 128-element randomized phased array with a 10.3 cm diameter at the opening and a radius of curvature of 7.2 cm (*f*-number 0.7) was used for all experiments (Chaplin *et al.*, 2018). Code was implemented to turn off outer elements of the transducer to electronically reduce its aperture (Fig. 1). Experiments were performed at three different *f*-numbers: *f*/0.7, *f*/0.8, and *f*/1.0. The largest aperture, *f*/0.7, used the full array, and the smallest aperture, *f*/1.0, was closest in *f*-number to previous MR-ARFI experiments performed by our group using a single element transducer (Phipps *et al.*, 2019; Yang *et al.*, 2021) and used 62 of the 128 elements. We calibrated the transducer by measuring the instantaneous pressure vs time with a fiber optic hydrophone (FOH) (Precision Acoustics, Dorchester, UK) across a range of powers up to that needed to generate 4 MPa of PNP for each *f*-number configuration. Calibration curves were fit for both the PNP and peak positive pressure (PPP). For each *f*-number configuration, the hydrophone was moved to find the maximum voltage as measured by an oscilloscope (PicoScope 5000, Pico Technology, St Neots, UK) recording the hydrophone voltage signal from a 100 μs pulse. The time series of the 100 μs pulse was recorded for increasing driving amplitudes until approximately 4 MPa of PNP was measured with a sampling time step of 8 ns. The Fourier transform of these time series data were calculated to assess the amount of energy in the center frequency and second harmonic at the spatial peak pressure location.

### 2.2 Pressure field simulations

Simulations of the transducer were carried out using the k-Wave toolbox for matlab (Treeby *et al.*, 2012; Treeby and Cox, 2010). The known element locations were used to create a model of the transducer with each element containing approximately 350 (303–421, mean 355) finite sources and a 50 cycle transmit wave. Simulations were performed with a homogeneous medium with speed of sound *c* = 1500 m/s, density ρ = 1000 kg/m^3^, attenuation coefficient of 0.5 dB/cm/MHz, nonlinearity parameter B/A = 7.0, isotropic voxel size of 0.3 mm, and a time step of 60 ns. The full time series for the simulation was recorded in a 91 × 41 × 41 voxel^3^ region around the focus. Because the focal pressure did not scale linearly with the input wave amplitude, an iterative process was used to match the focal pressure for each *f*-number. To match peak pressures between *f*-numbers, we first simulated the full array (i.e., *f*-number = 0.7), and then for f = 0.8 and f = 1, we iteratively scaled the source amplitude to match PNP at the focus within 1% of the full aperture. The PPP pressure maps were thresholded at 90% of the maximum PPP, and centroid shift was measured. The full width half maximum (FWHM) of each axis was determined for the focus ellipsoid. For every voxel in each simulation, the Fourier transform of the time series was calculated. The maximum amplitude around the center frequency of 650 kHz and the second harmonic of 1.3 MHz was measured.

### 2.3 Displacement simulations

Displacement simulations were adapted from Payne *et al.* (2015) and performed in matlab. The mean intensity of the FUS at each spatial location was calculated from the pressure simulations as $I=\mathrm{mean}(p^{2}/\rho c)$, where *p* is the pressure as a function of time, ρ is the density, and *c* is the speed of sound for the portion of the simulations after the FUS had arrived in every voxel. The acoustic radiation force was then calculated as F=2αI/c. The three-dimensional (3D) accumulated displacement (*w*) induced in the beam direction (*z*) by the radiation force in each simulated voxel was computed by $w=A^{\prime }g\left(r\right)F$, where $A^{\prime }g(r)=1/8\pi \mu (z^{2}/r^{3}+1/r)$ is a 3D Green's function with *r* representing the length of a vector from the internal steady state point source of the force to the displacement location, μ is estimated to be one-third of the Young's modulus assuming Poisson's ratio is 0.5, and *F* is the 3D force pattern. A Young's modulus of 20 kPa was used for the simulations. This Green's function was convolved with the force map via fast Fourier transform (FFT) of the function and the map, element-wise multiplication in the spatial frequency domain, and inverse FFT of the result. These equations assume elastostatic conditions are present, which is not the case during MR-ARFI, so the Green's function was weighted based on the velocity of the shear wave, $\beta =\sqrt{\mu /\rho }$, using a linearly decreasing function, which was 1 at the center of the Green's function to 0 at a radius that equals the FUS duration times the shear wave speed (Payne *et al.*, 2015). The peak displacement for each *f*-number configuration is reported.

### 2.4 MR-ARFI

We used MR-ARFI to map the displacement with three different *f*-numbers (*f*/0.7, *f*/0.8, *f*/1.0) in phantoms. The transducer was positioned vertically in a clinical 7 T MR scanner (Philips Achieva 7 T, Philips Healthcare, Best, Netherlands) with a water filled bubble for coupling. An agar-graphite (1% agar, 4% graphite) phantom was used for sonications. A spin echo MR-ARFI sequence was used for data acquisition [echo time (TE) = 16 ms, repetition time (TR) = 500 ms, MEG amp = 40 mT/m, MEG duration = 8 ms, 2 × 2 mm^2^ voxels with a 4 mm thick slice]. Ten ms-long sonications for each *f*-number were performed with matched input power for each element and with matched estimated focal pressure. The slice was perpendicular to the beam propagation direction and positioned at the geometric focus based on the distance from the transducer face as measured from a volumetric T1 image. Displacement maps were calculated from the ARFI data. A circle with a radius of 4 pixels inside the phantom but away from the focus was used to correct for phase background errors by subtracting off the mean phase in this region from the image.

Finally, *in vivo* MR-ARFI was performed in a non-human primate (NHP). All experiments were approved by the Institutional Animal Care and Use Committee of Vanderbilt University. The transducer was placed above the shaved head of the NHP and coupled with a water filled bubble and ultrasound gel. The transducer was aimed at the center of the brain but not targeted to a specific region. A two-dimensional (2D) MR-ARFI slice was acquired at the focus location such that the long axis of the beam would be visible. The same sequence that was used in phantoms was used with an extended TR of 1000 ms to reduce the risk of heat deposition. Sonications were 4.5 ms in duration, and a 3 ms MEG was used for MR-ARFI. To correct for background phase *in vivo*, we selected the region around the focus in the brain in the ARFI image to correct for phase offsets. Sonications were performed with the full array (*f*/0.7) and the smallest aperture configuration (*f*/1.0). The full array used a 5 MPa pulse, and the reduced array used a 4 MPa pulse based on the hydrophone calibration. Based on previous work through *ex vivo* skulls and simulations (Phipps *et al.*, 2019), we estimate 39% transmission at 650 kHz, which corresponds to focal pressure in the brain of 1.95 and 1.56 MPa for the two configurations.

## 3. Results

### 3.1 Generating matched negative pressure with reduced apertures

When outer elements were turned off, the transducer required a higher amplitude to reach the same PNP as measured with a hydrophone, scaling with the number of elements used for each configuration. Each configuration generated free field pressure expected to generate detectable displacements with MR-ARFI. In simulations, the focus location, as measured by the centroid of voxels above 90% of the maximum pressure, shifted toward the transducer by a small amount (1.0 mm) when using *f*/1.0 compared to *f*/0.7 (Table 1). As expected, the focus size increased with *f*-number with a larger FWHM in both the axial and lateral beam directions. The focus size in the axial direction in particular increased from 8.9 to 17.9 mm in simulations for the full array to the most reduced configuration (Table 1).

**Table 1.** Summary of pressure and displacement simulation results for three *f*-number configurations of the same transducer.

	*f*/0.7	*f*/0.8	*f*/1.0
PNP (MPa)	3.56	3.56	3.56
PPP (MPa)	4.11	4.27	4.57
Pressure FWHM (mm) (axial, lateral, lateral)	(8.9, 2.2, 2.2)	(11.6, 2.5, 2.4)	(17.9, 2.9, 2.8)
Intensity (W/cm^2^)	476.7	491.6	525.5
Maximum FFT amplitude around 650 kHz (F_0_)	2.66	2.70	2.80
Maximum FFT amplitude around 1300 kHz (2 F_0_)	0.33	0.36	0.61
2F_0_/F_0_	0.125	0.132	0.22
Displacement (μm)	3.2	4.1	5.9

### 3.2 Pressure matched simulations and hydrophone measurements show increased harmonic content

**Fig. 2.**
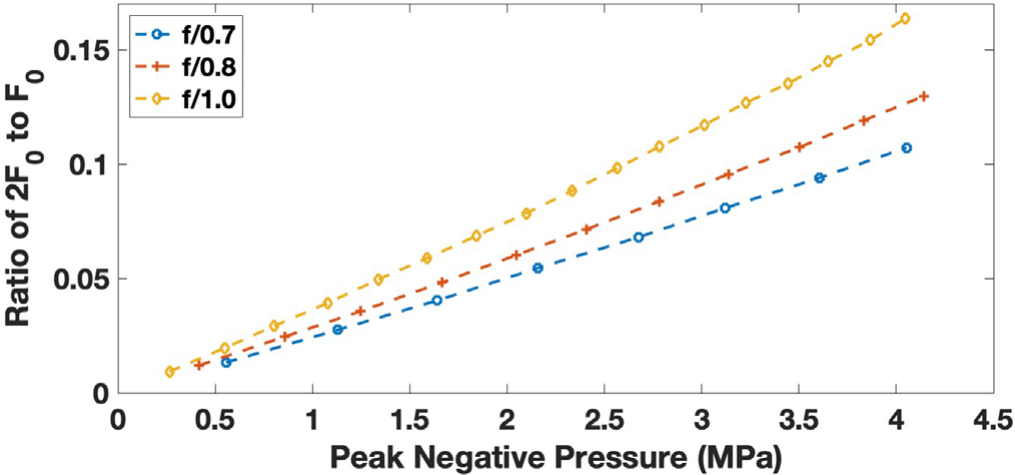
Hydrophone data comparing PNP vs the ratio of magnitude of the second harmonic (2 F_0_) to the center frequency (F_0_). Measurements of the pressure wave at the focus were performed for each *f*-number configuration, and the Fourier transform was used to measure the magnitude of the Fourier transform at the center frequency and second harmonic. At high pressure, there is more harmonic content in the wave within each *f*-number configuration. For larger *f*-numbers, we observed a higher ratio of harmonic content compared to the center frequency at similar pressures with the lowest ratio at *f*/0.7 (blue) increasing at *f*/0.8 (red) and *f*/1.0 (yellow).

Pressure matched simulations at *f*/1.0 had higher second harmonic content compared to simulations at *f*/0.7 (FFT magnitude of 0.61 and 0.33, respectively), while the FFT magnitude at the center frequency was 1.9 vs 2.0 (Table 1). The size and shift at both the center frequency and second harmonic were consistent with beam maps where the reduced-aperture *f*/1.0 case resulted in a larger beam that was shifted toward the transducer. As expected, the second harmonic had a small focus in all dimensions compared to the center frequency. Similar increases in harmonic content were seen in the fiber optic hydrophone data with a larger ratio of second harmonic to center frequency energy in the frequency content (Fig. 2).

### 3.3 Phantom studies detect increased displacement with increased f-number

**Fig. 3.**
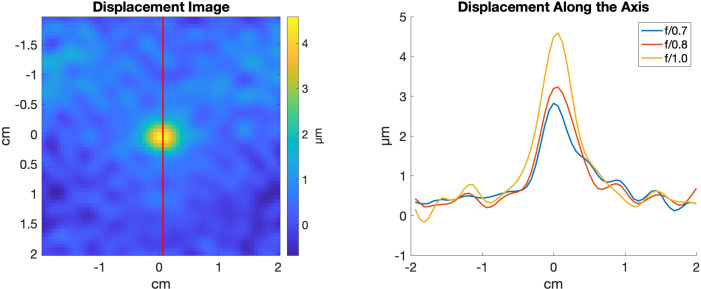
Phantom MR-ARFI displacement map showing the short axis view of the beam for *f*/1.0. The displacement is perpendicular to the image plane. The displacement along the beam (red line) is plotted to the right for each *f*-number configuration. Using pressure matched sonications, an increase in displacement was measured while using larger *f*-number apertures.

Using MR-ARFI, we measured increased displacement with increasing *f*-number. Figure 3 shows a representative scan where the measured displacement in a 3 × 3 pixel^2^ area at the focus increases from 2.6 μm at *f*/0.7 to 4.3 μm at *f*/1.0, while sonications used the same estimated free field PNP of 4.4 MPa.

### 3.4 In vivo imaging showed similar displacement at lower PNP with larger f-number

**Fig. 4.**
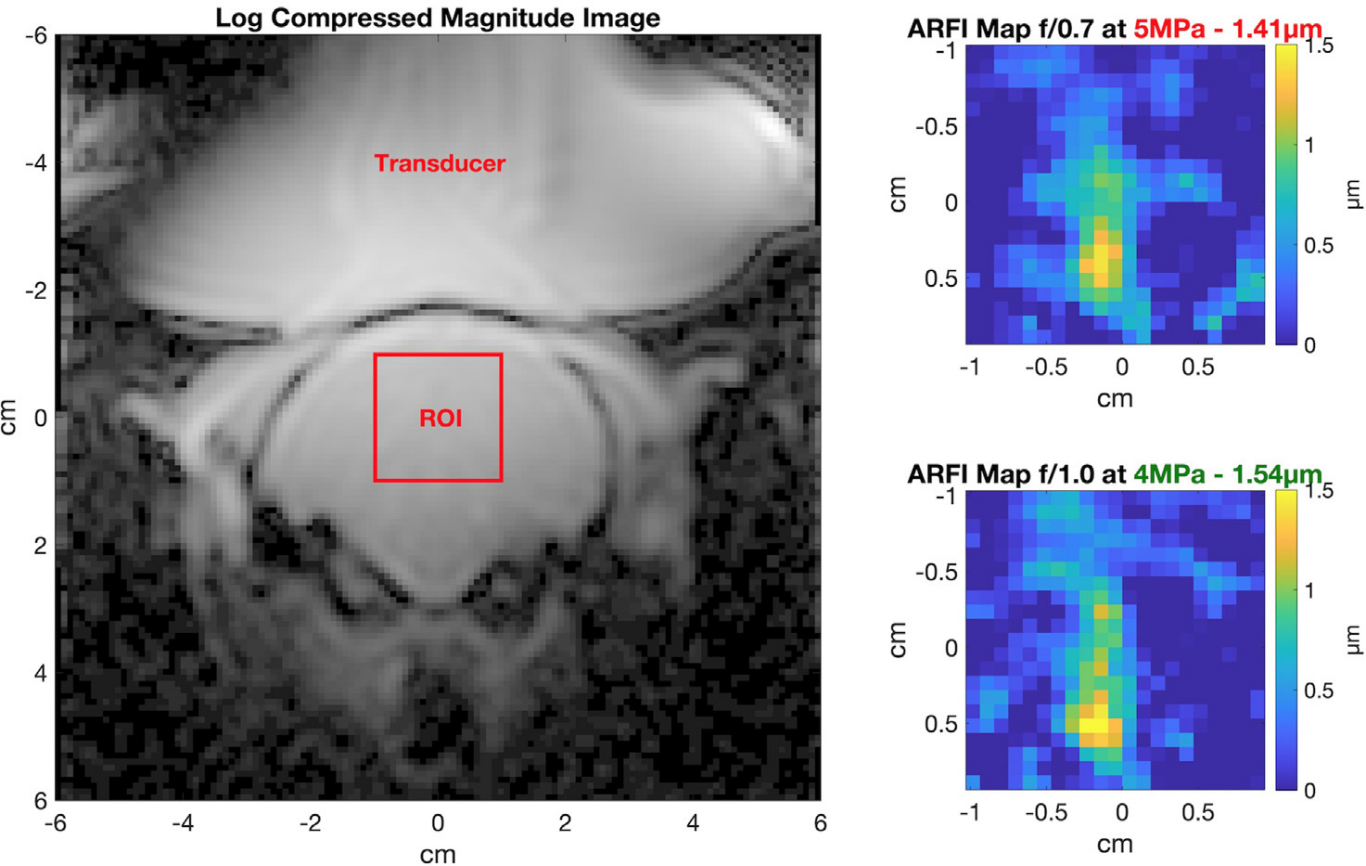
*In vivo* MR ARFI data. The magnitude image showing the positioning of the transducer over the NHP head and the region where the focus was located (left). A displacement of 1.41 μ m was measured with the full array (*f*/0.7) using 5 MPa of free field pressure (top right). With a reduced array (*f*/1.0), 20% less free field pressure was able to generate 1.54 μ m of displacement (bottom right).

In *in vivo* imaging, the *f*/1.0 beam generated the similar displacement as *f*/0.7 despite having 20% lower free field pressure (Fig. 4). The *in vivo* data were noisier than our phantom data, especially away from the focus region. The targeted region was in the center of the brain for these scans with the transducer placed above the NHP's head.

## 4. Discussion

Our results show that increasing the *f*-number of a FUS transducer enables it to generate the same displacement in MR-ARFI at lower pressure. Using lower energy can help prevent damage to brain tissue and the skull by reducing the potential for heating and mechanical damage. Evidence suggests that temperature changes in neurons can lead to altered activity (Wang *et al.*, 2014); thus, heating can lead to neurological changes that would confound the results of neuromodulation experiments.

Many factors could contribute to the observed increased displacement with reduced aperture. The increase in harmonic content observed in the simulations and hydrophone measurements, which is characteristic of nonlinear propagation, results in more absorption at higher frequency harmonics and thus a larger displacement. The direction of the emitted sound may also play a role because when the aperture is reduced, more pressure is output from the elements that are aligned with the displacement encoding direction (i.e., the main propagation direction), while the reduced aperture turns off the outer elements of the transducer that are less well-aligned. Finally, the larger focus size associated with the reduced-aperture configurations results in a broader intensity field at the focus, which may allow for larger peak displacements at the center of this field. This effect can be seen by exploring the simulation method (Payne *et al.*, 2015) used in this work. Convolving a larger force pattern with the same maximum force with the Green's function results in larger displacement.

Displacement in the phantom was larger than in *in vivo* experiments, which is consistent with key differences between phantom and *in vivo* experiments. The skull acts as a low pass filter that will reduce the magnitude of radiation force by reducing pressures at higher frequencies in the brain compared to a homogeneous phantom. Aberration induced by the skull may increase the focus size, which can reduce differences between each *f*-number configuration.

### 4.1 Heating and neuronal effects

Recent work has shown MR-ARFI pulses are safe and do not result in any histological changes (Gaur *et al.*, 2020). Similarly, two studies have shown in simulation and in macaques that MR-ARFI pulses result in <0.5 °C heating within the target brain tissue and minor heating of 2–3 °C within the skull (Ozenne *et al.*, 2020; Phipps *et al.*, 2019). The skull heating can result in an increase in the cortical brain temperature, and differences in skull morphology between individuals are known to affect how much energy is absorbed in the skull and transmitted to the brain (Boutet *et al.*, 2020). Temperature changes of less than 1 °C have been shown to affect the firing rates of preoptic single units, and these changes scaled with temperature for small temperature changes (Boulant, 1974). By lowering the needed energy to detect ARFI signal, we will lower the amount of heating that will occur, minimizing potential neuronal changes.

### 4.2 Focal shift and targeting accuracy

When increasing the *f*-number of our transducer, we observed a focal shift in the axial beam direction in both beam maps and simulations. This focal shift was smaller than the beam length in that direction. We also observed a widening of the beam at higher *f*-numbers. In phantom and *in vivo* imaging, these changes were difficult to observe with MR-ARFI, presumably due to the differences being on a similar size order of the minimum voxel size available for MR-ARFI scans. The acceptable beam size and shift will depend on which part of the brain is targeted as well as the relative focus size. For larger brain regions and larger beam sizes, these shifts can be an acceptable trade-off to reduce the needed energy. However, if these effects are much larger than the target region of the brain, it may not be possible to target that region with a reduced aperture. Additionally, the skull itself can result in changes to the beam location and shape of the focus (Kyriakou *et al.*, 2014), and the different beam paths for different *f*-numbers may not have the same aberration. These issues will be more prominent in a thicker human skull.

### 4.3 Minimizing energy deposition

The principle of “as low as reasonably achievable” (ALARA) is generally considered when thinking about potential energy dose (Bevelacqua, 2010). If we need to localize the FUS beam to perform an experiment or treatments, the safest option is to minimize the amount of energy deposited in the subject during the sonication. By increasing the *f*-number of our transducer, we were able to decrease the needed intensity to detect the focus. Combining this method with other improvements in the sensitivity of MR-ARFI will allow for even less energy being needed to localize the beam.

As discussed in the Introduction, other methods have been reported that provide increases to the signal-to-noise ratio of MR-ARFI or allow for fewer sonications to be used, which can also reduce the risk of damage or confounding effects in combination with the technique presented here. The combination of increased *f*-number with optimized gradients may allow for even less energy to be used to localize the beam. Standard MR acceleration methods have previously been implemented with MR-ARFI, such as keyhole acceleration (Paquin *et al.*, 2013) and single shot echo-planar imaging (EPI) (Kaye *et al.*, 2011). These acceleration techniques allow for fewer sonications to be used to produce a full displacement map, which will minimize the total amount of deposited energy.

Use of a high frequency transmitter would be an alternate way to efficiently generate displacement while reducing the likelihood of cavitation. Such a solution is technically feasible but would likely require additional high frequency transmit elements compared with those used for clinical applications, which typically range from 220 to 650 kHz. The higher frequency pressure wave would absorb more in the skull and generate heat. Although these trade-offs could likely be managed, the method demonstrated here requires no additional hardware and maintains a single transmit frequency.

## 5. Conclusions

We demonstrated and characterized a method to more efficiently generate displacement during transcranial ultrasound procedures. Our findings reduce the pressure required to localize the beam and can improve the safety profile when localizing the ultrasound focus during transcranial ultrasound procedures.
